# Impact of polymorphisms in DNA repair genes XPD, hOGG1 and XRCC4 on colorectal cancer risk in a Chinese Han Population

**DOI:** 10.1042/BSR20181074

**Published:** 2019-01-15

**Authors:** Dexi Jin, Min Zhang, Hongjun Hua

**Affiliations:** 1 Department of Gastrointestinal Surgery, the Affiliated Wenling Hospital of Wenzhou Medical University, the First People’s Hospital of Wenling, Zhejiang, China; 2 Department of Gastroenterology, Shanxian Dongda Hospital, Shandong, China; 3 Department of Gastroenterology, Jinhua Central Hospital, Jinhua Hospital of Zhejiang University, Zhejiang, China

**Keywords:** Colorectal cancer, DNA repair genes, gene polymorphism

## Abstract

Background: This research aimed to study the associations between XPD (G751A, rs13181), hOGG1 (C326G, rs1052133) and XRCC4 (G1394T, rs6869366) gene polymorphisms and the risk of colorectal cancer (CRC) in a Chinese Han population. Method: A total of 225 Chinese Han patients with CRC were selected as the study group, and 200 healthy subjects were recruited as the control group. The polymorphisms of XPD G751A, hOGG1 C326G and XRCC4 G1394T loci were detected by the RFLP-PCR technique in the peripheral blood of all subjects. Results: Compared with individuals carrying the XPD751 GG allele, the A allele carriers (GA/AA) had a significantly increased risk of CRC (adjusted OR = 2.109, 95%CI = 1.352–3.287, *P*=0.003). Similarly, the G allele (CG/GG) of hOGG1 C326G locus conferred increased susceptibility to CRC (adjusted OR = 2.654, 95%CI = 1.915–3.685, *P*<0.001). In addition, the T allele carriers (GT/TT) of the XRCC4 G1394T locus have an increased risk of developing CRC (adjusted OR = 4.512, 95%CI = 2.785–7.402, *P<*0.001). The risk of CRC was significantly increased in individuals with both the XPD locus A allele and the hOGG1 locus G allele (adjusted OR = 1.543, 95%CI = 1.302–2.542, *P*=0.002). Furthermore, individuals with both the hOGG1 locus G allele and the XRCC4 locus T allele were predisposed to CRC development (adjusted OR = 3.854, 95%CI = 1.924–7.123, *P*<0.001). The risks of CRC in XPD gene A allele carriers (GA/AA) (adjusted OR = 1.570, 95%CI = 1.201–1.976, *P*=0.001), hOGG1 gene G allele carriers (CG/GG) (adjusted OR = 3.031, 95%CI = 2.184–4.225, *P*<0.001) and XRCC4 gene T allele carriers (GT/TT) (adjusted OR = 2.793, 95%CI = 2.235–3.222, *P*<0.001) were significantly higher in patients who smoked ≥16 packs/year. Conclusion: Our results suggest that XPD G751A, hOGG1 C326G and XRCC4 G1394T gene polymorphisms might play an important role in colorectal carcinogenesis and increase the risk of developing CRC in the Chinese Han population. The interaction between smoking and these gene polymorphisms would increase the risk of CRC.

## Introduction

Colorectal cancer (CRC) is currently the third most common malignancy worldwide and ranks fourth in cancer-related mortalities [1]. Together with economic bloom, improvement in quality of life, changes in dietary patterns and environmental deterioration, the incidence of CRC is sharply increasing in developing countries including China [1]. According to the registration data collected from the National Central Registry of China, 3,763,000 new CRC cases (2,157,000 for male and 1,606,000 for female) and 1,910,000 cancer deaths (1,111,000 for male and 800,000 for female) were estimated from 2009 to 2011 [2]. The exact mechanisms underlying colorectal carcinogenesis remain unknown despite the epidemiological data indicating that numerous factors might contribute to the etiology of CRC, including high rates of red meat consumption, tobacco use, alcohol intake, lack of exercise and family history [3]. However, these conventional risk factors do not fully account for all cases, especially in young subjects, who often do not have any of these factors. In addition, family history of tumors significantly increased susceptibility to CRC, which showed that genetic factors might be related to CRC etiology, similar to the etiology of any other major malignancy [4,5].

A common type of genetic variation in the genome, known as single-nucleotide polymorphism (SNP), has been found to be associated with susceptibility to cancer [6]. Many of these polymorphisms are found in the genes that regulate potentially oncogenic pathways [6]. The DNA repair pathways play critical roles in maintaining genome integrity, and a diminished capacity to repair DNA lesions predisposes individuals to an increased susceptibility to cancer [7,8]. Individuals with CRC have been shown to have a lower DNA repair capacity [9,10]. We hypothesize that genetic polymorphisms in DNA repair genes may affect DNA repair capacity and increase the risk of CRC in a specified population. There are multiple DNA repair genes, each dealing with specific DNA damage [7]. Xeroderma pigmentosum group D (XPD), 8-oxoguanine DNA-glycosylase 1 (hOGG1) and X-ray repair cross-complementing protein 4 (XRCC4) are among the key DNA repair genes; XPD is thought to be involved in the nucleotide excision repair, hOGG1 is implicated in repairing oxidatively damaged DNA and DNA single-strand breaks, and XRCC4 primarily addresses DNA double-strand breaks, repaired by homologous and nonhomologous end-joining recombination (NHEJ) [11–13].

To obtain a comprehensive estimate of the putative influence of the genetic polymorphisms of these genes on CRC risk, XPD G751A, hOGG1 C326G and XRCC4 G1394T polymorphisms in CRC patients were detected in this case–control study in a sample of the Chinese population with the aim of providing a theoretical basis for the treatment and prognosis of the disease.

## Materials and methods

### Patient characteristics

This based case–control study was conducted from August 2014 until October 2017. A total of 225 consecutive CRC patients (140 males, 85 females; aged 53.1 ± 11.2 years) who underwent surgical resection at the Gastrointestinal Surgery, the Affiliated Wenling Hospital of Wenzhou Medical University were enrolled in the present study as the observation group, whereas 200 age- and sex-matched controls (118 males, 82 females; aged 49.7 ± 11.2 years) were individuals who received health screening at Tongde Hospital of Zhejiang. Subjects in the observation group were sporadic CRC patients without a family history of CRC. All individuals enrolled in the present study had no chronic diseases including diabetes mellitus, hypertension, cardiovascular and cerebrovascular disease, chronic kidney disease or other systemic diseases. All patients with CRC did not receive radiotherapy or chemotherapy before surgery. The data collected included sex, age, smoking and alcohol intake habits, tumor location, T stage and grade, lymph node status, distant metastases, and neoadjuvant chemotherapeutic treatment. Informed consent was obtained from all subjects. The study was approved by the Ethics Committee of the Affiliated Wenling Hospital of Wenzhou Medical University.

### DNA extraction and gene polymorphism detection

**Figure 1:**
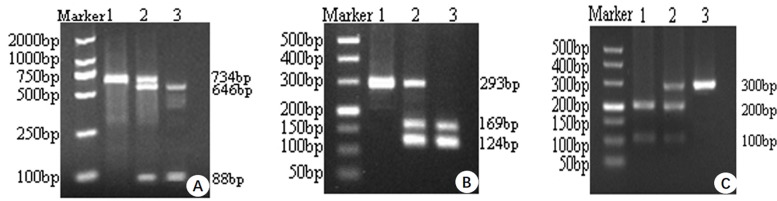
Detection of XPD, hOGG1 and XRCC4 polymorphisms through PCR/FFLP (**A**) lane1: XPD GG genotype; lane 2: XPD GA genotype; lane 3 XPDAA genotype. (**B**) lane 1: hOGG1 CC genotype; lane 2: hOGG1 CG genotype; lane 3: hOGG1 GG genotype. (**C**) lane 1: XRCC4 GG genotype; lane 2: XRCC4 GT genotype; lane 3: XRCC4 TT genotype.

Peripheral venous blood (5 ml) was obtained from all subjects in the morning, while they were in a fasting state. Genomic DNA was extracted using the DNeasy Kit (QIAamp DNA Blood Midi Kit, Qiagen, Cat#51104, Hilden, Germany) according to the manufacturers’ instructions. The extracted DNA was stored at −80°C for further use. The XPD G751A, hOGG1 C326G and XRCC4 G1394T polymorphisms were genotyped using a polymerase chain reaction-restriction fragment length polymorphism (PCR-RFLP) assay. The primers and restriction enzymes for determining XPD G751A, hOGG1 C326G and XRCC4 G1394T genotypes were shown in Table 1. The 25-μl PCR reaction system was used, containing 2.5 μl 10×Taq polymerase buffer solution, 2 μl magnesium chloride (2 mM), 2 μl dNTP mix (0.2 mM), 1 μl forward primer (10 pmol), 1 μl reverse primer (10 pmol), 2 μl genomic DNA (100 ng/μl), 0.5 μl DNA Taq polymerase enzyme and 14 μl distilled water. The PCR amplification conditions were as follows: denaturation at 94°C for 4 min, then 35 cycles of denaturation at 94°C for 30 s, annealing at 52°C for XPD, 60°C for hOGG1 and 59°C for XRCC4 for 30 s, and extension at 72°C for 40 s, followed by a final extension cycle at 72°C for 10 min. The PCR reaction products were treated with the appropriate restriction enzymes in 37°C water overnight, and the digested products were separated by electrophoresis in 2% agarose gels. Finally, an ultraviolet gel imager was used to visualize the electrophoretic results and determine sample genotypes. The PCR product information for the XPD G751A, hOGG1 C326G and XRCC4 G1394T variants were shown in Figure 1.

**Table 1:** Amplification primers and PCR amplification product information

Genotype	Primer sequence (forward and reverse)	PCR product	Restriction	Restriction products
XPD G751A	CCTCTCCCTTTCCTCTGTTC	734bp	PstI	GG:734bp; GA:734bp, 646bp, 88bp; AA:646bp, 88bp
	CAGGTGAGGGGGGACATCT			
hOGG1 C326G	ACTGTCACTAGTCTCACCAG	293bp	Fnu4HI	CC:293bp; CG:169bp,124bp; GG:124bp
	GGAAGGTGCTTGGGGAAT			
XRCC4 G139T	GATGCGAACTCAAAGATACTGA	300bp	HincII	GG:100bp; GT:200bp
	TGTAAAGCCAGTACTCAAACTT			TT:300bp

### Statistical analysis

Statistical analyses were conducted with the SPSS 18.0 software package (SPSS Inc, Chicago, IL). The heredity equilibrium was assessed by the Hardy–Weinberg test, and the differences in genotype frequencies of XPD G751A, hOGG1 C326G and XRCC4 G1394T between the CRC patient group and the control group were evaluated by the chi-square test. The linkage disequilibrium of gene polymorphisms was measured by *D*_0_, and the *r*^2^ value was calculated via the Haploview program (http://www.broad.mit.edu/mpg/haploview/). The correlations between polymorphism phenotype alleles and clinicopathological parameters, demographic variables and environmental factors were analyzed by the chi-square test or the Fisher exact test. The odds ratios (OR) with the 95% confidence interval (CI) were calculated to analyze the strength of the association of polymorphism phenotype alleles with CRC risk. Bonferroni adjustments were made for *P*-values for the results of any SNP by multiplying the number of SNPs tested for the gene. Receiver operating characteristic (ROC) curve analysis was performed to determine the optimal cut-off value to divide the smoking status between two groups. All *P*-values were two-sided, and *P<0.05 was considered to indicate statistical significance.

## Results

### Population characteristics

At baseline, no statistically significant differences between the study and control groups were noted regarding demographics (age and sex), BMI, smoking and alcohol status (Supplementary Table S1). The clinical diagnosis/staging of CRC was performed according to the Union for International Cancer Control (UICC) classification, and 74 patients with CRC received neoadjuvant chemotherapeutic treatment that lasted for 2 months. Among the CRC patients, 49.33% of lesions (111 cases) occurred in the colon, and 50.67% of lesions (114 cases) occurred in the rectum. There were 11 cases (4.89%) of T1 stage, 54 cases (24.00%) of T2 stage, 111 cases (49.33%) of T3 stage and 49 cases (21.78%) of T4 stage. A total of 77 cases (34.22%) had no lymphocyte metastases, denoted as N0, 36 cases (34.2%) were N1, 28 cases (12.44%) were N1a, 30 cases (13.33%) were N1b, 30 cases (13.33%) were N1 and 24 cases (10.67%) were N2. Regarding the tumor stages, 66 cases (29.33%), 102 cases (45.33%), 31 cases (13.78%) and 26 cases (11.56%) had stages I, II, III and IV disease, respectively. Forty-eight CRC patients (21.33%) had distant metastases, and the rest (177 cases, 78.67%) did not show signs of remote metastases.

### XPD, hOGG1, XRCC4 gene polymorphisms and CRC risk

The genotypic frequencies of polymorphisms were ascertained in a balanced state in the Han Chinese population, based on the Hardy–Weinberg equilibrium (*P*>0.05). Table 2 summarizes the associations of gene polymorphisms (XPD, hOGG1 and XRCC4) with CRC. For the XPD G751A polymorphism, our results revealed that individuals carrying the A allele (AG+AA) were associated with a significantly increased risk of CRC (OR = 2.118, 95%CI = 1.341–3.356, *P*=0.001; adjusted OR = 2.109, 95%CI = 1.352–3.287, *P*=0.003). Our results also suggested that the G allele carriers (CG+GG) of the hOGG1 C326G polymorphism were predisposed to CRC development, (OR = 1.71, 95%CI = 1.09–2.69, *P*=0.019). Similarly, a significant association between the XRCC4 G1394T polymorphism and susceptibility to CRC was observed in this case–control study (OR = 4.476, 95%CI = 2.741–7.360, *P*<0.001; adjusted OR = 4.512, 95%CI = 2.785–7.402, *P*<0.001) (Table 2).

**Table 2:** Distribution of XPD751, hOGG1, XRCC4 polymorphisms and CRC risk

Gene and genotype	CRC group (*n*=225)	Control group (*n*=200)	HWE*χ^2^*	HWE *P*-value	Crude OR (95%CI)	*P*-value	Adjusted OR (95%CI)^*^	*P*-value
XPD G751A								
GG	164 (72.89%)	167 (83.50%)	1.62	0.20	1	–	1	–
GA	56 (24.89%)	33 (16.50%)			1.728 (1.040–2.877)	0.025	1.705 (1.028–2.698)	0.034
AA	5 (2.22%)	0			–	–	–	–
Allele								
G	378 (84.00%)	367 (91.75%)			1	–	1	–
A	72 (16.00%)	33 (8.25%)			2.118 (1.341–3.356)	0.001	2.109 (1.352–3.287)	0.003
hOGG1 C326G								
CC	88 (39.11%)	138 (69.00%)	0.01	0.91	1	–	1	–
CG	114 (50.67%)	56 (28.00%)			3.192 (2.060–4.954)	<0.001	3.158 (2.057–4.865)	<0.001
GG	23 (10.22%)	6 (3.00%)			6.011 (2.208–17.226)	<0.001	6.024 (2.231–17.351)	<0.001
Allele								
C	290 (64.44%)	332 (83.00%)			1	–	1	–
G	160 (35.56%)	68 (17.00%)			2.694 (1.932–3.777)	<0.001	2.654 (1.915–3.685)	<0.001
XRCC4 G139T								
GG	137 (60.89%)	176 (88.00%)	0.81	0.37	1	–	1	–
GT	76 (33.78%)	24 (12.00%)			4.068 (2.375–7.008)	<0.001	4.054 (2.368–7.054)	<0.001
TT	12 (5.33%)	0 (0.00%)			–	–	–	–
Allele								
G	350 (77.78%)	376 (94.00%)			1	–	1	–
T	100 (22.22%)	24 (6.00%)			4.476 (2.741–7.360)	<0.001	4.512 (2.785–7.402)	<0.001

* ‘OR’ adjusted by age, sex, alcohol and smoking; CI, confidence interval; HWE, Hardy–Weinberg equilibrium.

### The joint effect of XPD, hOGG1 and XRCC4 gene polymorphisms on CRC risk

Gene loci of XPD G751A, hOGG1 C326G and XRCC4 G1394T were not interlinked (*D*_0_, 0.005; *r*^2^, 0.002). According to the frequencies of the relevant genotypes for XPD G751A, hOGG1 C326G and XRCC4 G1394T, a simultaneous occurrence of the XPD locus A allele, the XRCC4 locus T allele and the hOGG1 locus C allele was deemed risk-free. Given that all three polymorphisms, XPD G751A, hOGG1 C326G and XRCC4 G1394T, were not found in any individual, we examined the pairwise joint effect of variant alleles of XPD G751A, hOGG1 C326G and XRCC4 G1394T on CRC risk (Table 3). Interestingly, individuals carrying the XPD locus A allele and OGG1 locus G allele showed an increased CRC risk (OR = 1.85, 95%CI = 1.331–2.584, *P*<0.001; adjusted OR = 1.543, 95%CI = 1.302–2.542, *P*=0.002). Similarly, the combined effect of the hOGG1 locus G allele and the XRCC4 locus T allele was to increase susceptibility to CRC development (OR = 2.461, 95%CI = 1.826–3.317, *P*<0.001; adjusted OR = 3.854, 95%CI = 1.924–7.123, *P*<0.001). However, no association between the concurrence of the XPD locus A allele and the XRCC4 locus T allele and the risk of CRC was found (OR = 0.794, 95%CI = 0.572–1.101, *P*=0.150; adjusted OR = 0.654, 95%CI = 0.787–1.054, *P*=0.196).

**Table 3:** Combined effects of XPD751, hOGG1, XRCC4 and CRC risk

XPD G751A	hOGG1 C326G	XRCC4 G139T	Crude OR (95%CI)	*P*-value	Adjusted OR (95%CI)*	*P*-value
GG	CC	–	1	–	1	–
GA/AA (A)	CG/GG (G)	–	1.854 (1.331–2.584)	<0.001	1.543 (1.302–2.542)	0.002
–	CC	GG	1	–	1	–
–	CG/GG(G)	GT/TT(T)	2.461 (1.826–3.317)	<0.001	3.854 (1.924–7.123)	<0.001
GG	–	GG	1	–	1	–
GA/AA(A)	–	GT/TT (T)	0.794 (0.572–1.101)	0.150	0.654 (0.787–1.054)	0.196

* ‘OR’ adjusted by age, sex, alcohol and smoking; CI, confidence interval.

### Stratification analysis by smoking for the three gene polymorphisms and CRC risk

**Figure 2:**
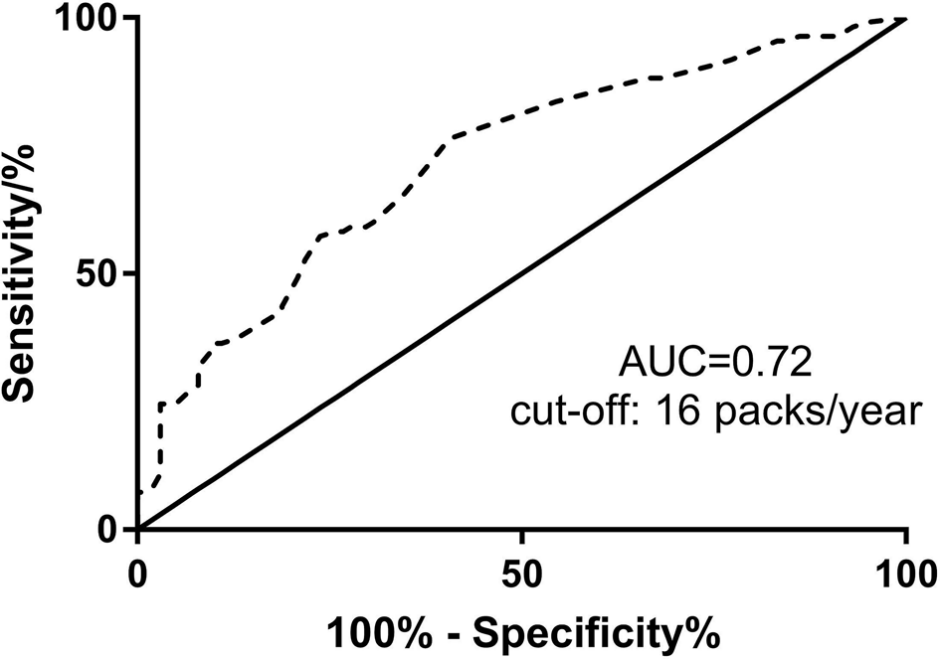
ROC curve for smoking status The area under the ROC curve was 0.72. The optimal cut-off value was 16 packages/year.

We further investigated the associations between the XPD, hOGG1, XRCC4 gene polymorphisms and CRC risk in a study stratified by smoking status. ROC curve analysis was performed to discriminate smoking status. As shown in Figure 2, the area under the ROC curve was 0.72 and the optimal cut-off value was 16 packages/year. The results are shown in Table 4. The risk of CRC in the XPD gene A allele carriers (GA/AA) was significantly higher in patients who smoked ≥16 packs/year (OR = 2.838, 95%CI = 1.490–5.433, *P*=0.001; adjusted OR = 1.570, 95%CI = 1.201–1.976, *P*=0.001); the risk of CRC in the hOGG1 gene G allele carriers (CG/GG) increased significantly (OR = 9.938, 95%CI = 5.061–19.694, *P*<0.001; adjusted OR = 3.031, 95%CI = 2.184–4.225, *P*<0.001); the risk of CRC in the XRCC4 gene T allele carriers (GT/TT) also increased significantly (OR = 19.695, 95%CI = 7.903–51.271, *P*<0.001; adjusted OR = 2.793, 95%CI = 2.235–3.222, *P*<0.001).

**Table 4:** Stratification analysis by smoking status for the three gene polymorphisms and CRC risk

Genes	Smoking	Genotypes	CRC (*n*=225)	Control (*n*=200)	Crude OR (95%CI)	*P*-value	Adjusted OR (95%CI)*	*P*-value
XPD G751A	<16	GG	102 (45.33%)	88 (44.00%)	1.00 (Ref)		1.00 (Ref)	
		GA/AA	12 (5.33%)	11 (5.50%)	0.941 (0.366–2.427)	0.891	0.972 (0.568–1.401)	1
	≥16	GG	62 (27.56%)	79 (39.50%)	1.00 (Ref)		1.00 (Ref)	
		GA/AA	49 (21.78%)	22 (11.00%)	2.383 (1.490–5.433)	0.001	1.570 (1.201–1.976)	0.001
hOGG1 C326G	<16	CC	62 (27.56%)	61 (30.50%)	1.00 (Ref)		1.00 (Ref)	
		CG/GG	52 (23.11%)	38 (19.00%)	1.346 (0.751–2.418)	0.287	1.146 (0.874–1.483)	0.354
	≥16	CC	26 (11.56%)	76 (38.00%)	1.00 (Ref)		1.00 (Ref)	
		CG/GG	85 (37.78%)	25 (12.50%)	9.938 (5.061–19.694)	<0.001	3.031 (2.184–4.225)	<0.001
XRCC1 G139T	<16	GG	92 (40.89%)	85 (42.50%)	1.00 (Ref)		1.00 (Ref)	
		GT/TT	22 (9.78%)	14 (7.00%)	1.452 (0.660–3.218)	0.317	1.176 (0.813–1.541)	0.413
	≥16	GG	45 (20.00%)	94 (47.00%)	1.00 (Ref)		1.00 (Ref)	
		GT/TT	66 (29.33%)	7 (3.50%)	19.695 (7.903–51.271)	<0.001	2.793 (2.235–3.222)	<0.001

Smoking (package/year).

### Stratification analysis by sexes for the three gene polymorphisms and CRC risk

As there was significant difference in the incidence of CRC between males and females, we studied the impact of sexes on the relationship between the three gene polymorphisms and CRC risk [14]. As shown in Table 5, we observed no association between the XPD, hOGG1, XRCC4 gene polymorphisms and risk of CRC in patients stratified by sexes.

**Table 5:** Stratification analysis by sexes for the three gene polymorphisms and CRC risk

Genes	Sexes	Genotypes	CRC (*n*=225)	Control (*n*=200)	Crude OR (95%CI)	*P*-value	Adjusted OR (95%CI)*	*P*-value
XPD G751A	male	GG	99 (44.00%)	95 (47.50%)	1.00 (Ref)			
		GA/AA	41 (18.22%)	23 (11.50%)	1.711 (0.919–3.197)	0.070	1.255 (0.962–1.562)	0.095
	female	GG	65 (28.89%)	72 (36.00%)	1.00 (Ref)			
		GA/AA	20 (8.89%)	10 (5.00%)	2.215 (0.903–5.523)	0.056	1.405 (0.950–1.841)	0.088
hOGG1 C326G	male	CC	74 (32.89%)	95 (16.44%)	1.00 (Ref)			
		CG/GG	66 (29.33%)	23 (14.67%)	3.684 (2.024–6.742)	<0.001	1.694 (1.354–2.051)	<0.001
	female	CC	14 (6.22%)	43 (3.11%)	1.00 (Ref)			
		CG/GG	71 (31.56%)	39 (15.78%)	5.592 (2.581–12.269)	<0.001	2.628 (1.651–4.466)	<0.001
XRCC1 G139T	male	GG	85 (37.88%)	102 (18.99%)	1.00 (Ref)			
		GT/TT	55 (24.44%)	16 (12.22%)	4.125 (2.117–8.120)	<0.001	1.704 (1.365–2.022)	<0.001
	female	GG	52 (23.11%)	74 (11.56%)	1.00 (Ref)			
		GT/TT	33 (14.67%)	8 (7.33%)	5.870 (2.356–15.088)	<0.001	1.950 (1.453–2.370)	<0.001

Smoking (package/year).

## Discussion

CRC is one of the most commonly diagnosed malignancies in East Asia and many other parts of the world. With the development of modern gastrointestinal endoscopy technologies and the establishment of surveillance protocols for individuals at high risk, more cases are diagnosed at the early stages, providing more opportunities for curative surgical resection [15]. However, due to the high malignant potential of CRC, approximately 40% of the surgically cured patients experience cancer recurrence within 5 years. CRC usually arises through a multistep carcinogenic process involving the accumulation of numerous genetic and epigenetic changes in oncogenes and suppressor genes, leading to dysregulation of multiple signaling pathways, which disrupt the cell cycle and the balance between cell proliferation and cell death [16,17]. In recent years, considerable interest has arisen in genetic factors that seem to modulate individual susceptibility to multifactorial diseases, characterized by SNPs that can be associated with a predisposition to and a high risk for development of carcinogenesis upon exposure to similar environmental and lifestyle factors [18,19].

Thousands of DNA lesions occur in cells, resulting from exposure to a variety of endogenous and exogenous chemical and physical agents [7]. If not correctly repaired, the accumulation of DNA damage could lead to global genomic instability and DNA rearrangements, which are commonly found in the majority of cancer cells. Efficient repair of this damage helps to maintain DNA stability [8]. There is certain evidence that deficiencies in the DNA repair capacity predispose individuals to an increased susceptibility to cancer [8]. Not surprisingly, individuals with CRC have been reported to have a lower DNA repair capacity. XPD, a prototypical 5’-3’ translocating DNA helicase, is part of the transcription factor (TF)IIH complex that is essential for signaling events triggering transcription, cell cycle checkpoints and DNA damage repair [20]. In the present study, we found that the XPD G751A gene polymorphism was associated with an increased risk of CRC, which was consistent with previous studies of other malignancies [21]. The XPD G751A polymorphism caused the amino acid substitution from Lys to Gln, which was closely associated with the impaired DNA repair capacity and thus predisposed individuals to an increased susceptibility to cancer [22,23]. Nevertheless, no significant association was found between the XPD G751A polymorphism and CRC susceptibility in the study from a Polish population [24]. Several reasons may contribute to the inconsistent result. First, Caucasians and Asians have different genetic backgrounds. The minor allele frequency of the XPD G751A genotype in control subjects was significantly lower in Chinese studies than that in Polish studies. Second, cancer is a complex disease affected by interactions between genetic, epigenetic and environmental factors. These factors may modify CRC risk in a distinct way in different populations. In other words, the same genotype of SNP might play an opposite biological role in tumor development in different ethnic groups. It is extremely valuable to address this issue specifically in a Chinese population and it is of great help to determine the relation in larger populations.

Base excision repair (BER) is an important DNA repair pathway of base damage and single-strand breaks caused by X-rays, oxygen radicals or alkylating agents. One of the key enzymes in the BER pathway is human hOGG1 [25]. Genetic variants of *hOGG1* may affect the expression and function of the OGG1 protein, thus contributing to the risk of cancer [26]. Ser326Cys is the most extensively studied *hOGG1* variant, and the Cys326 allele is increasingly reported to be associated with an increased risk of cancer [22,27,28]. Our results indicated that the hOGG1 C326G gene polymorphism and the CRC susceptibility were significantly correlated: the G allele carriers had a higher risk of developing CRC, which corresponded well with the previous study by Park et al. [29]. Sliwinski et al. [24] detected the XPD G751A and hOGG1 C326G gene polymorphisms in a Polish population and found no association between the locus gene polymorphisms and the risk of CRC. This discrepancy can be also explained by the different ethnicities studied or the complex underlying genetic architecture or multifactorial genetic factors of CRC as indicated above. But in addition, we noticed that this study contained a very limited sample size, which may as well lead to the controversial finding.

XRCC4, located on chromosome 5q14.2, is an important DNA repair gene involved in the NHEJ pathway. XRCC4 directly interacts with Ku70/Ku80 and plays a central role in the precise end-joining of blunt DSBs [30]. It has been reported that inactivation of the *XRCC4* gene causes growth defects, premature senescence, inability to support V(D)J recombination, late embryonic lethality accompanied by defective lymphogenesis, and defective neurogenesis manifested by extensive apoptotic death of newly generated post-mitotic neuronal cells [31]. Mutations in the coding region of this gene might result in a more deficient NHEJ capacity and increase cancer risk. In the present study, we found that the XRCC4 G1394T gene polymorphism had a substantial association with the increasing risk of CRC, in which T allele carriers had a higher risk of CRC (adjusted OR = 4.512, 95%CI = 2.785–7.402, *P*<0.001). In consistent with this observation, the T allele homozygotes for this SNP has been demonstrated to exhibit a defective DNA repair capacity and correlate with higher chromosome aberration frequency.

We also examined the joint effects of XPD, hOGG1 and XRCC4 gene polymorphisms on the risk of CRC development and found that the AG/AA genotype of XPD together with the GG genotype of hOGG1 increases the rate of developing CRC. In addition, a combination of the G allele of hOGG1 and the T allele of XRCC4 conferred higher CRC susceptibility. Identifying patients at risk of developing CRC through genotyping may allow a more personalized approach to moderate the risk of CRC development. We further investigated whether tobacco smoking behavior affect the interactions between the polymorphisms and CRC risk. After stratifying the subjects by smoking degree, the A allele carriers of XPD G751A, the G allele carriers of hOGG1 C326G and the T allele carriers of XRCC4 G1394T were associated with a significantly higher risk of CRC. Tobacco has been recognized as a major risk factor for CRC. It contained a large amount of toxic and harmful substances and can produce free radicals, which can lead to DNA damage [32]. There was great potential that an increase DNA damage and a reduced DNA repair capacity coordinately increase tumor susceptibility. Despite incidence of CRC was higher in males, we observed no association between the three gene polymorphisms and risk of CRC in the stratified analysis based on sexes. This finding was plausible as XPD G751A, hOGG1 C326G and XRCC4 G1394T were common autosomal variants, by which the relationship with CRC risk should not be differed by sexes.

Our findings might be helpful in early detection of CRC through identifying population at risk and the clinical monitoring value is greater among individuals with a severe smoking level. However, these findings should be treated with caution because of the relatively modest sample size and heterogeneity. Well-designed studies with a larger scale and more ethnic groups are needed to validate the risk factor.

## Conclusion

Our study provided compelling evidence that XPD G751A, hOGG1 C326G and XRCC4 G1394T gene polymorphisms were associated with the susceptibility to developing CRC in a Chinese Han population. Although there were some existing limitations in the study regarding the diversity or ethnicity of samples, our findings provided further insights into the pathogenesis of CRC.

## Supporting information

**Table S1:** Characteristics of patients with CRC and controls

## Abbreviations

BER: base excision repair
CI: confidence interval
CRC: colorectal cancer
hOGG1: human 8-oxoguanine DNA-glycosylase 1
NHEJ: nonhomologous end-joining recombination
ROC: receiver operating characteristic
SNP: single-nucleotide polymorphism
XPD: Xeroderma pigmentosum group D
