# Supplementary material for: Impact of polymorphisms in DNA repair genes XPD, hOGG1 and XRCC4 on colorectal cancer risk in a Chinese Han Population
Source: Biosci Rep. 2019 Jan 15;39(1):BSR20181074. doi: 10.1042/BSR20181074 (PMC6331672; doi:10.1042/BSR20181074)
Supplement: Supplementary file 1 [file bsr20181074_Supp1.pdf]

Table S1 Characteristics of patients with CRC and controls

Parameters	CRC group (n=225)	Control group (n=200)	χ^2-value	*p*-value
Age (years)			6.018	0.111
20~40	7(3.00%)	8(4.00%)		
41~60	101(44.89%)	102(51.00%)		
61~80	112(49.78%)	90(45.00%)		
≥81	5(2.22%)	0(0.00%)		
Gender			0.461	0.497
Male	140(62.22%)	118(59.00%)		
Female	85(37.78%)	82(41.00%)		
BMI (kg/m^2)	25.84±6.55	25.79±6.83	0.077	0.939
Smoking status			0.058	0.810
Yes	111(49.33%)	101(50.50%)		
No	114(50.67%)	99(49.50%)		
Alcohol consumption			2.702	0.1
Yes	54(24.00%)	35(17.50%)		
No	171(76.00%)	165(82.50%)		
